# Supplementary material for: Association between oral microbiome and five types of respiratory infections: a two-sample Mendelian randomization study in east Asian population
Source: Front Microbiol. 2024 Apr 10;15:1392473. doi: 10.3389/fmicb.2024.1392473 (PMC11039966; doi:10.3389/fmicb.2024.1392473)
Supplement: Supplementary file 1 [file Data_Sheet_1.zip › Supplementary Materials/MR plots of saliva/Tonsillitis/s__Porphyromonas_sp000467855_mgs_2773/leave_one_out.pdf]

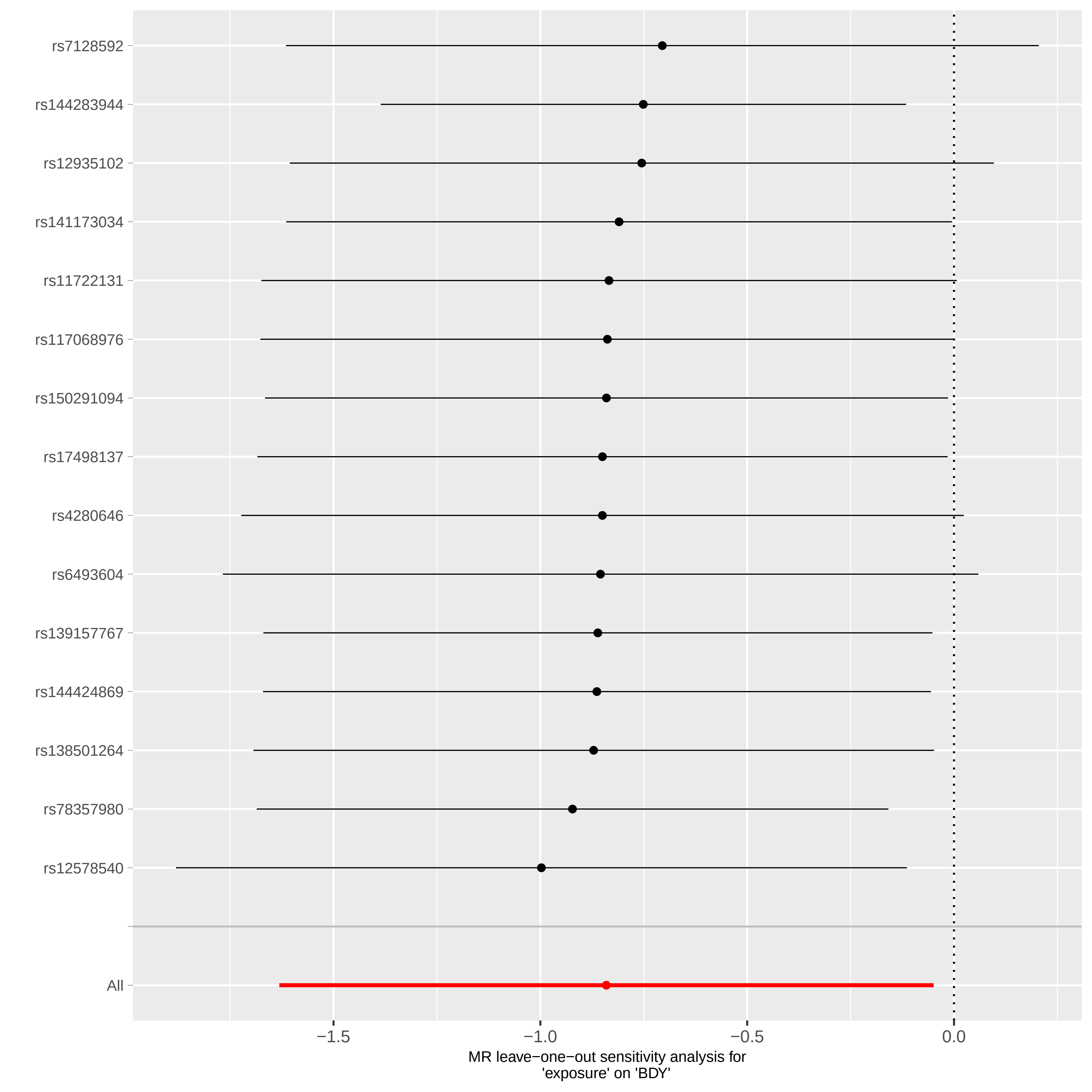
rs7128592
rs144283944
rs12935102
rs141173034
rs11722131
rs117068976
rs150291094
rs17498137
rs4280646
rs6493604
rs139157767
rs144424869
rs138501264
rs78357980
rs12578540
All
−1.5
−1.0
−0.5
0.0
MR leave−one−out sensitivity analysis for
'exposure' on 'BDY'
